# Comparing auditory vs. virtual reality relaxation in reducing dental anxiety

**DOI:** 10.3389/fdmed.2025.1625411

**Published:** 2025-11-17

**Authors:** Caley Mintz, Kenneth J. Spolnik, Drashty Paresh Mody, Ygal Ehrlich, George J. Eckert, Leslie Flowers, Stuart M. Schrader

**Affiliations:** 1 Clinician, Endodontist, Indianapolis, IN, United States; 2 Department of Endodontics, Indiana University School of Dentistry, Indianapolis, IN, United States; 3 Department of Biomedical and Applied Sciences, Indiana University School of Dentistry, Indianapolis, IN, United States; 4 Department of Biostatistics and Health Data Science, Indiana University School of Medicine, Indianapolis, IN, United States; 5 Student Wellness and Communications, Indiana University School of Dentistry, Indianapolis, IN, United States

**Keywords:** endodontics, dental anxiety, educational, nonpharmacological interventions, clinical outcomes, patient-reported outcomes

## Abstract

**Background:**

Up to 20% of American adults experience dental anxiety, creating a psychological and physiological barrier to starting, completing, and/or finishing dental treatment. There is a clear need for novel approaches to mitigate chair-side anxiety, especially for endodontic treatment appointments. This study aimed to investigate two nonpharmacological dental anxiety management approaches: (1) A brief auditory-alone relaxation (ABR) period and a brief virtual reality relaxation (VRR) period, and their hypothesized effects on patients' perceived dental anxiety and physiological biometric scores.

**Materials and methods:**

Fifty-eight participants who needed nonsurgical root canal treatment were assigned randomly to two groups: ABR or VRR. One group received earphones to listen to a guided, brief relaxation recording that incorporated conscious, diaphragmatic breathing and a guided body scan. The other group received Meta Quest 2 virtual reality headsets to listen and watch 360° inclusive and integrative experiences of ambient music, high-resolution graphic illustrations, and immersive scenery. The participants’ self-reported levels of anxiety were assessed before and after treatment after they completed the State Trait Anxiety Indicator (STAI-State & Trait) and visual analog scale (VAS) scales. Additionally, biometric traits such as heart rate (HR), systolic blood pressure (SBP), and diastolic blood pressure (DBP) were measured before (T0), during (T1), and after (T2) treatment.

**Results:**

Both VRR and ABR significantly decreased anxiety, as reported by the STAI-State questionnaire (*p* = <0.001 for both) and the STAI-Trait questionnaire (*p* = 0.025 ABR; *p* = <0.001 VRR), throughout the appointment. The self-reported VAS scores also were reduced significantly from before to after treatment (*p* = <0.001 for both ABR and VRR). The heart rate also decreased from before to after treatment in both groups (*p* = 0.019 for the ABR group, *p* = 0.026 for the VRR group). Changes in blood pressure showed mixed results. No significant differences in the blood pressure outcomes were found between the two groups.

**Conclusion:**

This is the first study to investigate the effects of ABR and VRR on dental anxiety. Both the ABR and VRR groups presented significant reductions in anxiety, pain, and heart rate after treatment. Our study demonstrated that nonpharmacological techniques, such as ABR and VRR, can be valid, noninvasive approaches to reduce anxiety before dental treatment, specifically endodontic therapy. However, given the small cohort in this study, it will be necessary to reproduce the methods with a larger cohort and different types of ABR and VRR applications to confirm the effects of nonpharmacological interventions on reducing endodontic dental anxiety.

## Introduction

### Background

Anxiety, a debilitating and difficult sensation characterized by tension and worried thoughts, contributes to increased blood pressure (1) and other physiological changes. Approximately 20% of U.S. adults experience anxiety, especially in a dental clinical setting (2). Dental anxiety can present with psychological (e.g., feelings of fear, disembodiment, disengagement) and physiological (e.g., increased heart rate and pulse or lower oxygen levels) signs that adversely impact the treatment process (3, 4). There is also emerging evidence on the putative effect of sociodemographic factors on the experience of dental anxiety (5).

To mitigate dental anxiety, nonpharmacological methods (meditation, music, and, more recently, virtual reality) have started to show promising patient management outcomes (reduced chair time and increased patient satisfaction) (2, 4, 6, 7). These relaxation response training courses show similar improvements in endodontic patients' overall thoughts (8). More specifically, music, video relaxation, and brief relaxation have also decreased dental anxiety in endodontic patients (4, 9).

VR provides a high-resolution, inclusive, and vividly simulated visual and auditory 3D sense of “immersion” (10, 11). Immersion equates to a state of presence, which gives the user a sense of interactive control over the digital world (12). The altered presence can, in turn, attenuate the brain's regulatory mitigation of anxiety and pain by altering a user's ability to sense visual and auditory experiences (13). More specifically, VR relaxation (VRR) has been shown to be effective in reducing overall relaxation and anxiety in dental patients (14). Additionally, VR over a five-minute period reduces dental anxiety prior to generalized dental procedures (15).

Both virtual reality relaxation (VRR) and auditory-assisted brief relaxation (ABR) have demonstrated value in mitigating anxiety preoperatively in dental patients. However, there are presently no comparative ABR or VRR patient anxiety assessments conducted during endodontics-related dental visits. Therefore, the purpose of this study was to compare the efficacy of ABR and VRR before root canal therapy on patient-reported [State Trait Anxiety Inventory (STAI)] anxiety levels and clinical anxiety-related traits (blood pressure and heart rate). We hypothesized that ABR and VRR both would have a positive effect on a patient's perceived dental anxiety and subsequently positively impact their secondary physiological anxiety traits of blood pressure and heart rate, and that the effects would be greater for VRR than ABR because of its immersive characteristics.

## Methods and materials

This study was approved by Indiana University's Institutional Review Board (17053). and the Indiana University School of Medicine's Clinical Translational Science Institute. Its outcomes are posted with the ClinicalTrials.gov NCT05720897 (registered 09/04/23), prospectively registered before first enrollment.

### Materials

#### Technological instruments

There were two intervention materials used in this study:

Auditory Brief Relaxation (ABR) and Virtual Reality Relaxation (VRR). The ABR protocol utilized prerecorded layered music performed by a Board-certified health and wellness coach/qualified teacher of mindfulness-based stress reduction for patients. This recording consists of suggestive elements for reducing chair-side anxiety in real time by having the patient focus on scanning their body for points of tension and relaxing through diaphragmatic breathing techniques.

**Figure 1:**
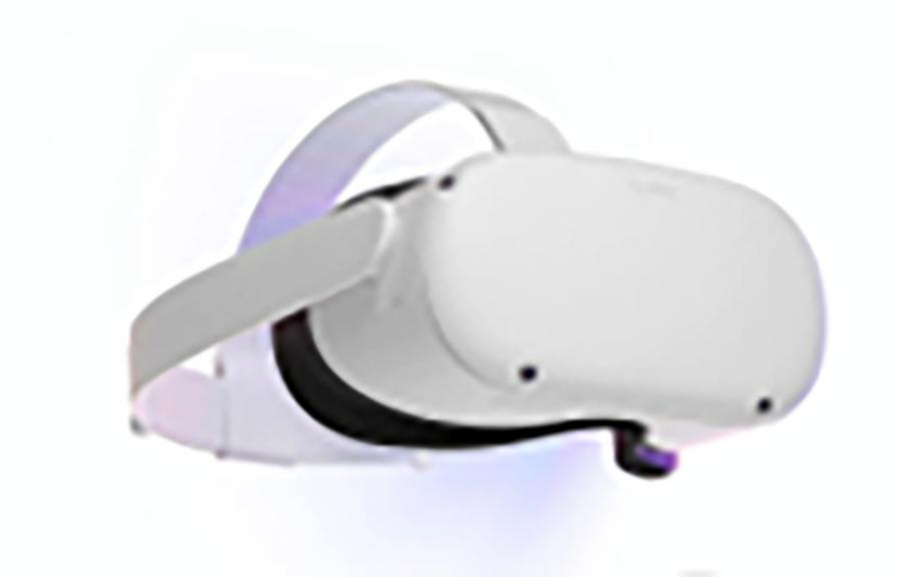
Meta Quest 2 headset utilized in the virtual reality relaxation (VRR) intervention.

The VRR experience involved a non-motive and non-interactive experience using the Meta Quest 2 headset (Figure 1). In 2020, the Meta Quest 2 VR headset functions in terms of both wired and wireless connection capacities, and two touch controllers. There are built-in speakers with volume adjusters and four cameras around the headset to track the user's body positioning. Meta Quest 2 has a fast-switch LCD display. Importantly, the headset is equipped with a “guardian system” with a “passthrough” feature to scan safety around the area and prevent the user from suddenly colliding with objects around it (16). Patients were trained to ask how to place and adjust the headset for comfort and volume. Controllers were not distributed to the study participants so that VRR was more uniform and to avoid an interactive gamification experience. Research has shown no difference in perceived pain relief, for example, between interactive and noninteractive VR experiences (17). The standardized app on the headset was the NatureTrek App (*GreenerGames*). The participants could select from 11 themed environments, with each 8-min session ranging from a savannah to a beach to a meadow. The influence of different virtual environments on patient self-reported and biometric variables indicative of dental anxiety was not included in the assessment.

### Clinical parameters

Patient biometrics, blood pressure (BP), and heart rate (HR) data were collected to compare the changes in dental anxiety before and after postoperative endodontic treatment. A calibrated Omron blood pressure cuff was used to measure the patient's BP and HR. Patient-reported measures: Patients were asked to rate their anxiety before, during, and after treatment via the Visual Analog Scale (VAS) (7, 18), Wong-Baker Scale [validated in (19)] (0–10 pictorial scale, 0 = no anxiety and 10 = extreme anxiety) (Figure 3), and the State-Trait Anxiety Indicator (STAI) scale (1, 2, 4, 20). STAI is a 40-item questionnaire with a four-item response set that ranges from “very much” to “not at all,” which correlates to a numerical value. Higher scores indicate heightened anxiety (Figures 4a,b).

### Experimental design

**Inclusion criteria:** Candidates for the study were selected from patients scheduled in a graduate and undergraduate endodontic clinic for evaluation of nonsurgical root canal therapy (NSRCT) and treatment of irreversible pulpitis (IRP). The age range for patients was 18–90 years, with sufficient mental capacity to independently provide informed consent. The participants were proficient in English and had no visual or hearing impairments that would interfere with listening with earphones or the use of a VR headset.

**Figure 2:**
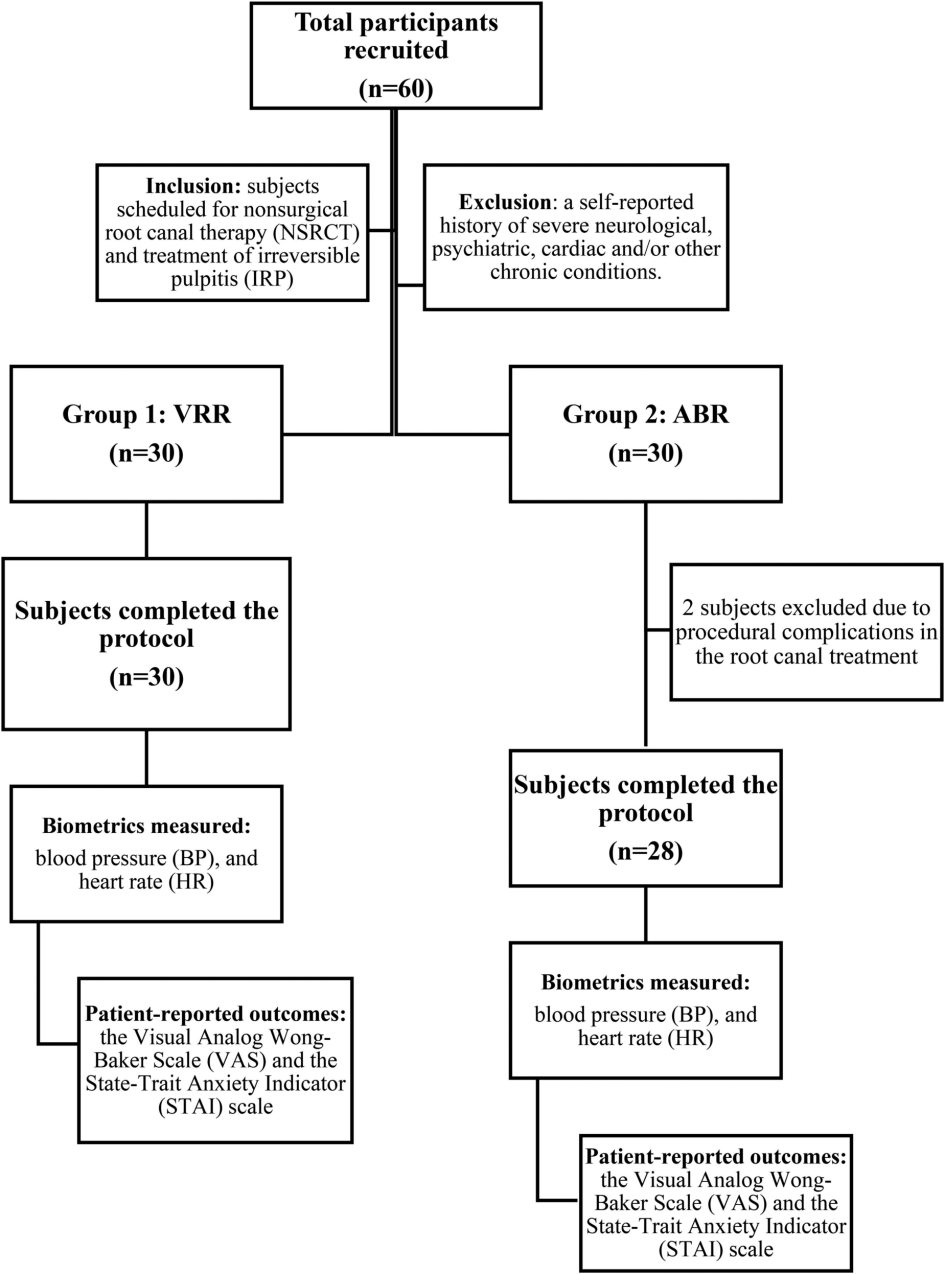
Workflow of the experiment design of this study. Initially, there were 60 participants enrolled, but only 58 completed the study.

**Exclusion criteria:** a self-reported history of vertigo, severe motion sickness, severe psychiatric disease, a medical history of seizures, concussions, severe neurological conditions, visual and hearing impairments, or a cardiac pacemaker or defibrillator. Additionally, patients who required nitrous oxide sedation, pharmacologic anxiolytics, or sedatives for treatment were also excluded (Figure 2).

**Figure 3:**
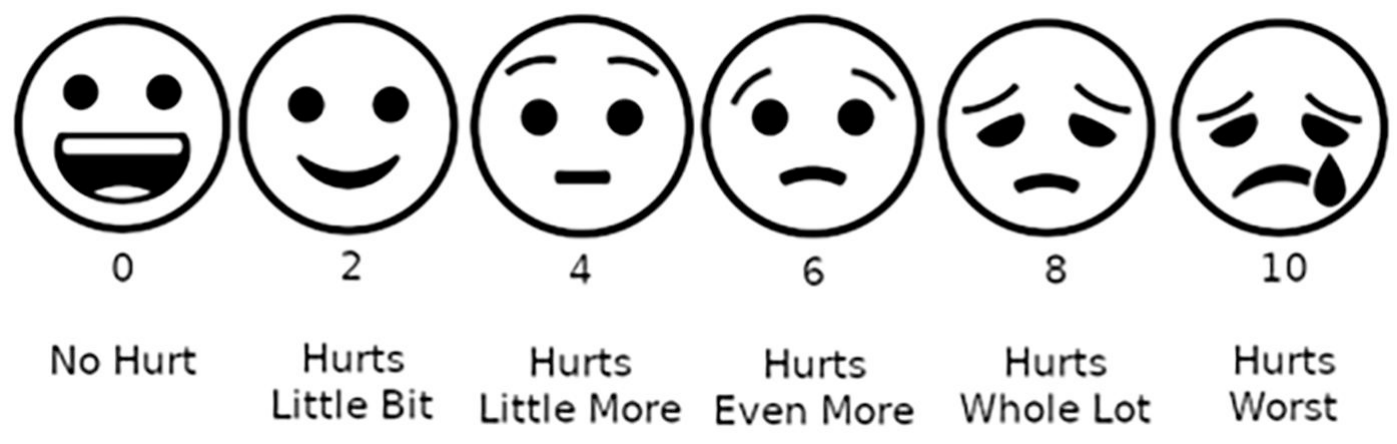
Adapted Wong–Baker scale (VAS) utilized for recording patients’ self-reported anxiety before, during and after treatment (image source: https://commons.wikimedia.org/wiki/File:Wong-Baker_scale_with_emoji.png).

**Figure 4:**
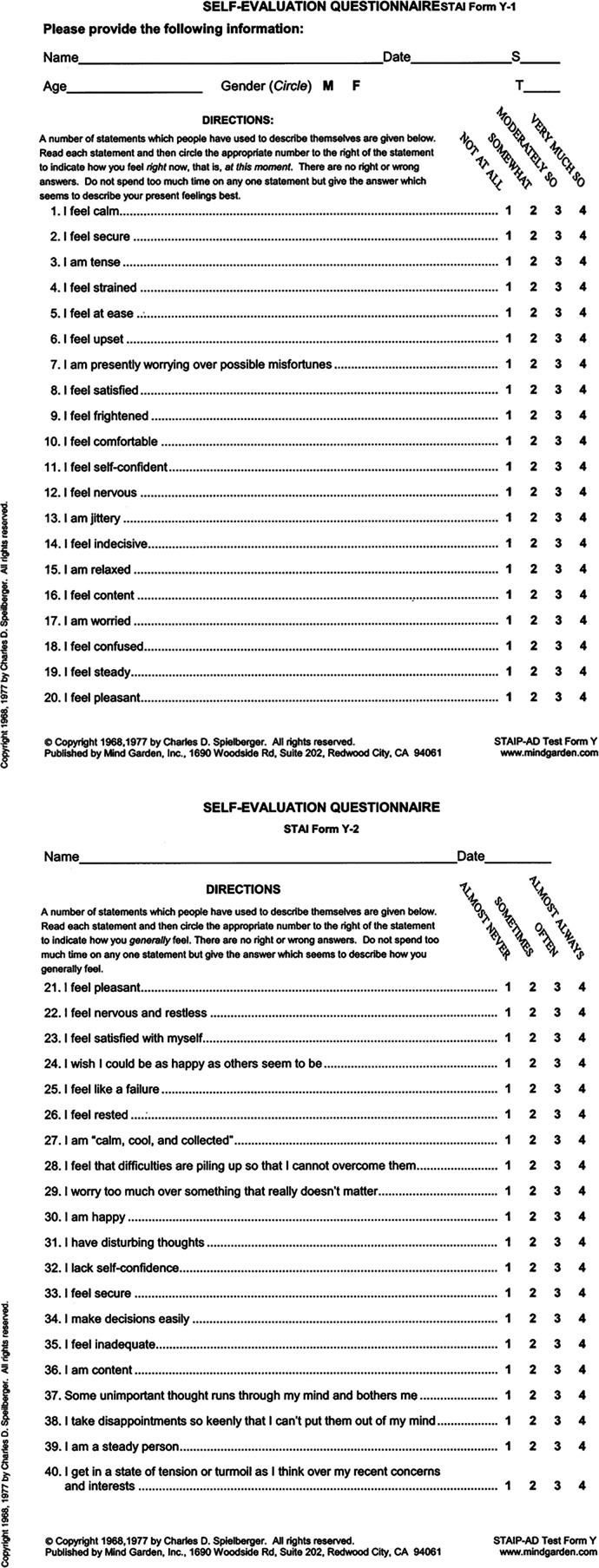
**(a)** State-trait anxiety indicator (STAI) scale—pg.1 utilized to record patients’ self-reported anxiety before, during and after treatment. **(b)** State-Trait Anxiety Indicator (STAI) scale—pg.2 utilized to record patients’ self-reported anxiety before, during and after treatment.

### Procedure

Patients who met the inclusion criteria for IRP and provided medical history and consent for NSRCT were randomized into ABR or VRR groups. The randomization list was generated using R statistical software to randomly assign the participants equally two the two groups, using a block randomization to ensure group balance throughout study enrollment. The student researcher and a Clinical Research Coordinator for the IUSD, Oral Health Research Institute, worked together to administer the randomized assignments. This is not a blind study in such that both the student researcher and the Clinical Research Coordinator knew what type of intervention was provided to which participant.

Both the investigator and clinical research coordinator knew the type of intervention provided to participants. The clinical investigator was not blinded to the biometric measurements as these parameters were recorded during the patient appointment as per the patient care protocols established at the Indiana University School of Dentistry (IUSD). Before administering local anesthetics, patients were given the appropriate headphones as part of either the ABR or VRR group. Both interventions were performed for 8 min.

Patient biometric and self-reported data were collected at three different timepoints: a) T0 (pre-endodontic treatment), b) T1 (approximately 10 min after the ABR or VRR interventions), and c) T2 (after completion of endodontic treatment and final radiographs).

Additionally, the amount of time from local anesthesia to patient dismissal was recorded for further analysis. No adverse effects were observed during the study.

### Statistical analysis

R statistical software was used to randomize 60 participants evenly between the ABR and VRR groups. This study was designed to have 80% power at a two-sided 5% significance level to detect changes within each group of 3.6 in patients' self-reported anxiety (STAI responses, effect size 0.55 via paired *t* tests) and differences of 5.0 between groups (effect size 0.77 via a two-sample *t* test), with 28 subjects per group completing the study. To account for a potential 5% dropout rate, 30 patients were enrolled per group.

Chi-square tests and two-sample t tests were used to compare the two intervention groups for differences in demographic characteristics. Owing to the nonnormality of the outcomes, Wilcoxon signed rank tests were used to test changes in the outcomes over time within each group, and Mann‒Whitney *U* tests were used to compare the outcomes between the two groups (ABR vs. VRR) at each time point (T0, T1 and T2). Exploratory analyses evaluated the associations of demographic characteristics with changes in the outcomes via cumulative logistic regression. A two-sided, 5% significance level was used for all tests. Analyses were performed via SAS version 9.4 (SAS Institute, Inc., Cary, NC, USA).

## Results

Two individuals dropped out of the ABR group because of procedural difficulties with an incomplete NSRCT during the single visit; thus, 28 ABR subjects and 30 VRR subjects completed the study.

### Study demographics

The mean ages for the ABR and VRR groups were 41.9 years/old and 45.9 years/old, respectively (range = 19–81 years across the groups). Among those in the ABR group, 64% were female, and 36% were male. In the VRR group, 43% were female, and 53% were male. In both groups, 57% identified as Caucasian. Additional race and ethnicity details are compiled in Table 1. Finally, the mean overall treatment time was 89 min, with a range from 34 min to 153 min (ABR average = 88.6 min, VRR average = 90.63 min). Demographics were not significantly different between the groups (*p* > 0.20).

**Table 1:** Patient demographics.

Demographic	Value	ABR (*N* = 28)	VRR (*N* = 30)
Gender Identity	Female	18 (64%)	13 (43%)
	Male	10 (36%)	16 (53%)
	Other	0 (0%)	1 (3%)
Race	American Indian/Alaskan Native	0 (0%)	1 (3%)
	Asian	1 (4%)	3 (10%)
	Black or African American	8 (29%)	5 (17%)
	Native Hawaiian or Other Pacific Islander	0 (0%)	1 (3%)
	White	16 (57%)	17 (57%)
	Other	3 (11%)	3 (10%)
Ethnicity	Non-Hispanic	23 (82%)	26 (87%)
	Hispanic	5 (18%)	4 (13%)
Age (years)	Mean (SD)	41.89 (18.23)	45.90 (17.41)
Treatment time (min)	Mean (SD)	88.68 (32.61)	90.63 (27.16)

Demographics were not significantly different between intervention groups.

### ABR vs. VRR intervention outcomes

#### Self-reported anxiety changes: ABR vs. VRR

The STAI evaluation was split into two categories: a) the STAI-S (“S” = present state, including the ability to focus primarily on the autonomic nervous system) and b) the STAI-T (“T” = trait, one's susceptibility and general state of anxiety) (21).

Overall, both intervention modalities contributed to changes in the mean STAI-S and STAI-T scores (Table 2). The range of patients' self-reported STAI-S and STAI-T scores ranged from “low” to “lower high” anxiety at T0 (preintervention). At T1, most of the scores were “low” to “moderate” and then decreased after treatment (T2). As demonstrated in Table 1, the patients from the ABR self-reported a decrease in the average STAI-S score from T0 (33.75) to T2 (27.39) (*p* < 0.001). These patients also reported a decrease in the average STAI-T score from T0 (31.86) to T2 (30.18) (*p* = 0.025). The study participants in VRR self-reported a significant reduction in the average STAI-S score from T0 (35.13) to T2 (29.37) (*p* < 0.001). These patients also reported a decrease in the average STAI-T score from T0 (33.77) to T2 (29.90) (*p* < 0.001). Additionally, the VRR group reported a significant decrease in the average VAS score from T0 (2.43) to T2 (1.17) (*p* < 0.001). In parallel, the ABR group also reported a significant decrease in the average VAS score from T0 (2.68) to T2 (0.96) (*p* < 0.001). The changes in the STAI-S (*p* = 0.858), STAI-T (*p* = 0.067), and VAS scores (*p* = 0.418) did not significantly differ between the intervention groups.

**Table 2:** Self-reported patient anxiety scores and secondary biometrics across the ABR (*n* = 28) and VRR (*n* = 30) groups.

Outcome	Time	Group	Mean	SD	Median	Q1	Q3	Min	Max
STAI-S	0	ABR	33.75	9.87	34.5	24	40	20	53
		VRR	35.13	10.64	33.5	28	42	20	57
	2	ABR	27.39	7.84	26	20	30.5	20	43
		VRR	29.37	9.01	26	22	37	20	48
STAI-T	0	ABR	31.86	10.94	30	23	37.5	20	61
		VRR	33.77	10.22	31.5	27	38	20	64
	2	ABR	30.18	11.55	26	20.5	39	20	64
		VRR	29.90	8.53	28	24	35	20	54
VAS	0	ABR	2.68	2.44	2	0.5	4	0	10
		VRR	2.43	2.05	2	1	4	0	8
	1	ABR	1.57	1.79	1	0	2	0	8
		VRR	1.73	2.41	0.5	0	3	0	9
	2	ABR	0.96	0.96	1	0	2	0	3
		VRR	1.17	1.97	0	0	2	0	7
SBP (mmHg)	0	ABR	134.68	15.63	134	122.5	145	105	171
		VRR	131.90	16.81	130.5	119	142	104	165
	1	ABR	132.50	18.74	128.5	121	143	103	181
		VRR	133.47	20.93	132	119	144	95	192
	2	ABR	142.64	20.03	138	126.5	160	113	184
		VRR	137.47	22.45	134.5	130	146	99	201
DBP (mmHg)	0	ABR	82.36	10.29	84.5	76	88.5	59	101
		VRR	81.83	10.65	78.5	75	89	67	108
	1	ABR	81.25	12.30	79	73.5	88	61	115
		VRR	77.80	15.39	76.5	69	87	46	111
	2	ABR	90.00	12.56	89.5	82	102	62	111
		VRR	85.97	15.48	85	76	96	59	137
Heart rate (bpm)	0	ABR	77.82	11.53	73.5	68.5	86	62	111
		VRR	76.97	11.89	75.5	70	82	57	111
	1	ABR	72.36	9.84	69.5	66	76.5	58	101
		VRR	73.30	9.67	71.5	66	80	58	95
	2	ABR	72.89	9.09	71	68	77.5	60	93
		VRR	72.17	10.90	74.5	61	79	56	93

No significant differences were found between intervention groups.

#### Biometric outcome (BP and HR) changes: ABR vs. VRR

The biometric outcomes are summarized in Table 2. In both groups, the average systolic blood pressure (SBP) increased from T0 to T2: ABR (T0 = 134. 68 ± 15.63, T2 = 142.64 ± 20.03, *p* = 0.018) and VRR (T0 = 131.90 ± 16.81, T2 = 137.47 ± 22.45, *p* = 0.014) (Table 2). The average diastolic blood pressure (DBP) increased from T0 to T2 within the ABR group (T0 = 82.36 ± 10.29, T2 = 90.00 ± 12.56, *p* = 0.002) but not significantly in the VRR group (T0 = 81.83 ± 10.65, T2 = 85.97 ± 15.48, *p* = 0.080) (Table 2). The average heart rate (bpm) decreased across both the ABR (T0 = 77.82 ± 11.53, T2 = 72.89 ± 9.09, *p* = 0.019) and VRR groups (T0 = 76.97 ± 11.89, T2 = 72.17 ± 10.90, *p* = 0.026) (Table 2). These changes were not significantly different between the ABR and VRR groups for SBP (*p* = 1.00), DBP (*p* = 0.232), or heart rate (*p* = 0.907).

#### Additional exploratory findings

Overall, demographic characteristics were not significantly associated with changes in the STAI-S, STAI-T, or VAS score. There were, however, a few significant associations with biometric outcomes. There was a significantly greater increase in the average heart rate (HR) from T1 to T2 for younger participants (*p* = 0.036): 19- to 29-year-olds increased from 74.5 bpm at T1 to 76.8 bpm at T2, 30- to 40-year-old patients maintained a steady average HR (T1 = 72.5, T2 = 72.9 bpm), while 41- to 58-year-olds had an HR of 71.5 bpm at T1, which slightly decreased to 68.2 bpm at T2, and 59–81-year-old subjects had a T1 HR of 72.4 bpm, which slightly decreased to 71.4 bpm at T2 (data not shown).

SBP (*p* = 0.046) and DBP (*p* = 0.014) changed differently by sex. Males increased from T0 to T1 (133.9–137.9 SBP and 80.5–82.1 DBP), whereas females decreased from T0 to T1 (132.3–128.8 SBP and 83.2–77.5 DBP) (data not shown).

There was an increase in HR with shorter treatment times (*p* = 0.028). Patients with the shortest appointment times (33–66 min) had an average HR of 71.5 bpm at T1, which slightly increased to 73.9 bpm at T2. The slightly longer appointments (67–92 min) resulted in an average HR of 71.6 bpm at T1, with a slight increase to 72.5 bpm at T2, whereas longer appointments lasting between 93 and 111 min recorded an average HR of 72.8 bpm at T1, which decreased to 70.5 bpm at T2 The longest appointments (112–153) min resulted in an average HR of 75.6 bpm at T1, which slightly decreased to 73.1 bpm at T2 (data not shown).

## Discussion

Dental anxiety is a difficult, multifaceted challenge that can affect up to one- fifth of the US adult population (2). Traditionally, many dentists, specifically endodontists, rely on anxiolytic or IV sedation and/or general anesthesia to reduce dental anxiety (22). However, some patients are beginning to prefer VR rather than the usual anesthetic treatment for managing their pain and anxiety (23). As such, our study is the first to demonstrate the beneficial use of nonpharmacological interventions involving VRR and ABR to mitigate patient anxiety during an endodontic appointment. Both VRR and ABR were found to reduce anxiety before and after endodontic treatment. Patient-reported outcomes (STAI-S, STAI-T and VAS) in both groups demonstrated a statistically significant decrease in patient anxiety throughout the appointment, with VRR reporting a higher decrease.

Additional exploratory analyses were performed to evaluate secondarily whether demographic characteristics were significantly associated with anxiety-related outcomes across and within intervention groups. Younger patients were found to have a greater increase in heart rate. These changes may have been due to the greater sympathetic nervous system response in younger patients than in older patients (24). Interestingly, males were found to have greater increases in SBP and DBP. Although biological sex differences are expected, one possible explanation for the differences in BP among our study cohort could be, in part, the age range. Blood pressure is typically lower in a cross section of female patients than in male patients, as men's blood pressure begins to increase steadily from the third to the seventh decade, whereas women's blood pressure increases abruptly during the postmenopausal stage (25). As such, the interrelationship between the sample sizes and of age and sex may have contributed to a lower female BP measurement.

Overall, the results from our study are consistent with the literature on the use of various VR applications to manage patient anxiety for a range of dental procedures. VR applications, more specifically, can function as a distraction to help patients further reduce their sense of anxiety (26). A recent study by Ghobadi et al. (27) used VR as a nonpharmacological intervention and reported a positive impact of VR in mitigating short-term anxiety (during the appointment) and as a putative long-term aid in lowering the likelihood of remembering stressful procedures (27). ABR applications, specifically relaxation music, significantly reduced SBP, DBP and HR during root canal treatment (28).

### Our study had several limitations

The age range of patients included in the study was from 18 to 90 years of age. The wide range of ages can contribute to confounding age-related differences in anxiety responses to stimuli within the study. Interestingly, the reduction in BP was not consistent across the intervention groups. Possible limiting variables for these BP results include the variability in the amount of anesthetic (epinephrine) used per patient and the variability, when essentially necessary, in transitioning from the supine to the upright seated position (29, 30). The lack of use of noise-cancelling headphones may have posed a limitation in the collection of patient-reported outcomes. Additional limitations may include that the investigator was not blinded to the intervention category of the patients. This was partially mitigated by having objective secondary biometric assessments of BP and HR coupled with subjective self-reported evaluations of participants' anxiety. Further, eight minutes of intervention potentially were not enough time for the desired effect across biometric and patient-reported outcomes. The longer timeframe of ABR or VRR in future studies could help patients feel more “immersed” in the calming. However, the length of time patients are within a fully immersive VR environment needs to be considered, as too much dopamine may negatively influence biometric and self-reported outcomes (31). The limited sample size is also an issue with exploratory analyses of demographic effects.

Although there is emerging evidence that using VRR (and ABR) reduces dental anxiety across different dental procedures, further investigation is necessary to examine its impact on psychosocial stressors within specific dental specialties (adult restorative care, complex oral surgery, and endodontics or periodontics). Comparing VRR and ABR techniques with more established anxiety management practices (such as music therapy, meditation, and nitrous oxide) and adjusting for confounding variables (local anesthesia carpules count, posture, duration of treatment and patient age) can be efficacious in expanding our understanding of nonpharmacological patient behavioral management techniques.

## Conclusion

ABR and VRR are nonpharmacologic approaches that endodontists may use to help patients manage and even decrease their anxiety beyond pre- and intraoperative medicative solutions. This study suggests further that technology-based nonmedication interventions (VRRs and ABRs) emerge as beneficial alternatives to mitigate patients' dental anxiety. These noninvasive techniques could be used increasingly for community endodontic practice, increasing the probability of a better patient experience.

## Data Availability

The original contributions presented in the study are included in the article/Supplementary Material, further inquiries can be directed to the corresponding author.
